# Trade-offs and synergies between yield, labor, profit, and risk in Malawian maize-based cropping systems

**DOI:** 10.1007/s13593-018-0506-6

**Published:** 2018-05-30

**Authors:** Adam M. Komarek, Jawoo Koo, Beliyou Haile, Siwa Msangi, Carlo Azzarri

**Affiliations:** International Food Policy Research Institute, Washington DC, USA

**Keywords:** Agricultural households, Cropping systems, Groundnut, Maize, Synergies, Trade-offs

## Abstract

Land degradation, population growth, and chronic poverty in Eastern and Southern Africa challenge the sustainability of livelihoods for smallholder farmers. These farmers often manage soils depleted of nutrients, apply limited amounts of mineral fertilizer, and take decisions about their cropping systems that involve multiple trade-offs. The rotation of cereals with legumes bears agronomic and ecological merit; however, the socio-economic implications of the cereal-legume rotation require a deeper understanding. This study explores the yield, labor, profit, and risk implications of different legume and mineral fertilizer practices in maize-based cropping systems in central Malawi. Our method involves coupling crop modeling and an agricultural household survey with a socio-economic analysis. We use a process-based cropping systems model to simulate the yield effects of integrating legumes into maize monocultures and applying mineral fertilizer over multiple seasons. We combine the simulated yields with socio-economic data from an agricultural household survey to calculate indicators of cropping-system performance. Our results show that a maize-groundnut rotation increases average economic profits by 75% compared with maize monoculture that uses more mineral fertilizer than in the rotation. The maize-groundnut rotation increases the stability of profits, reduces the likelihood of negative profits, and increases risk-adjusted profits. In contrast, the maize-groundnut rotation has a 54% lower average caloric yield and uses more labor than the maize monoculture with mineral fertilization. By comparing labor requirements with labor supply at the household scale, we show for the first time that the additional labor requirements of the maize-groundnut rotation can increase the likelihood of experiencing a labor shortage, if this rotation is undertaken by farm households in central Malawi. We demonstrate that risk and labor factors can be important when examining trade-offs among alternative cropping systems.

## Introduction

**Fig. 1:**
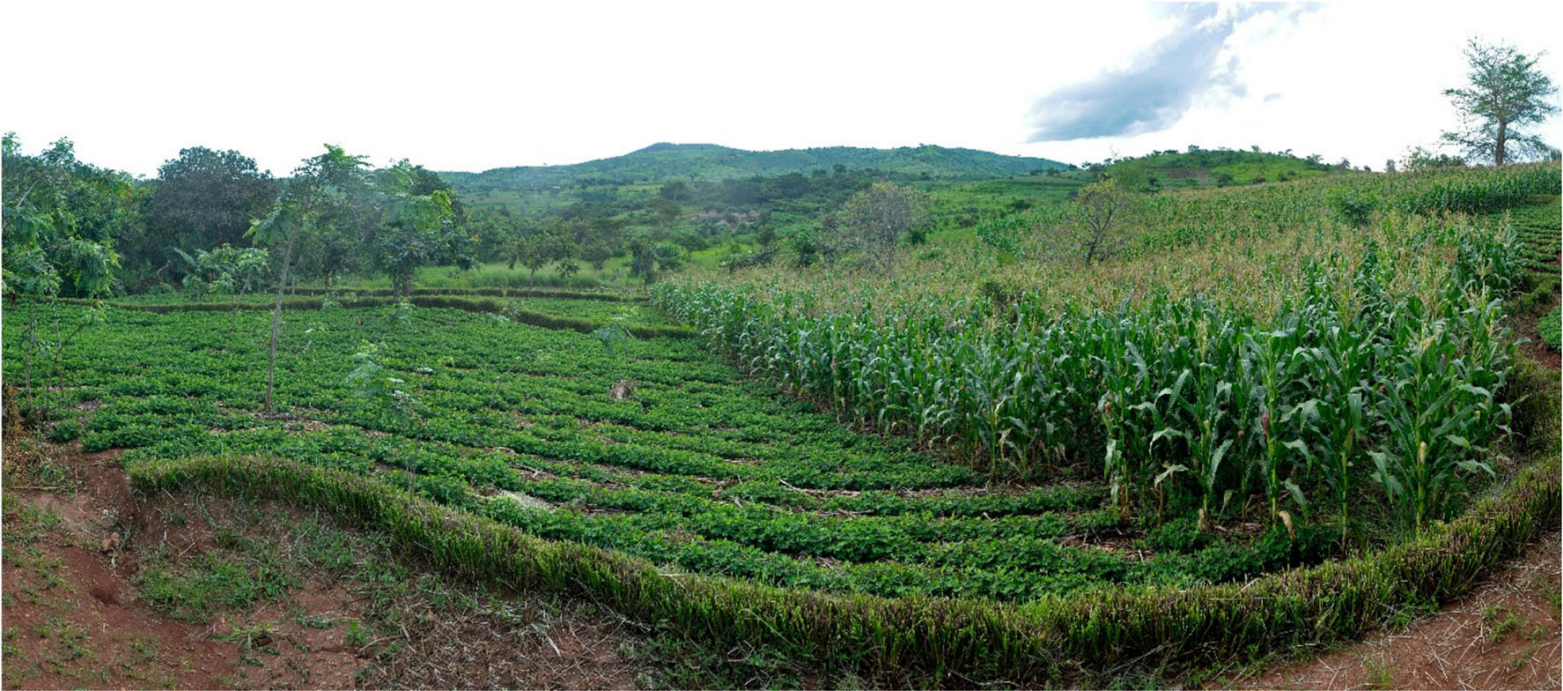
Malawian maize-groundnut rotation. Source: https://flic.kr/p/c7f7Vj. Photo credit: T. Samson/CIMMYT

Maize is the most commonly grown staple crop in Eastern and Southern Africa. Historically, cropping systems for smallholder farmers (hereafter farmers) in this region often included long fallows, which allowed soils to replenish their nutrients and in turn maintain crop productivity. In densely populated rural areas such as in central Malawi, high population pressure has strongly reduced the use of fallow, and farmers often practice the continuous cropping of maize (Thierfelder et al. 2013). This practice of maize monoculture in turn has reduced soil fertility. Focusing on improving the productivity of cropping systems is a long-standing, though still relevant, approach to improve the livelihoods of farmers who face declining soil fertility (Tittonell and Giller 2013). Combining legumes and mineral fertilizer (hereafter fertilizer) can help maintain farmer productivity and profits (Chianu et al. 2012; Onduru and Du Preez 2007; Chianu et al. 2011). In Malawi, maize-groundnut rotations with fertilizer (Fig. 1) are often more productive than maize monocultures (Thierfelder et al. 2013; Snapp et al. 2010; Ngwira et al. 2012).

Despite the often-observed productivity benefits of using alternative practices related to legumes and fertilizer in maize-based cropping systems in Eastern and Southern Africa (Droppelmann et al. 2017), multiple factors can influence their attractiveness. One factor is the availability of agricultural labor to meet the labor requirements for these practices (Ngwira et al. 2012). Practices are often developed and tested at the field scale, although farmers often encounter constraints to using these practices at the farm or household scale. This recognition of constraints has led to different initiatives, such as the Soil Health Consortia for Eastern and Southern Africa, seeking to identify the socio-economic feasibility of different technologies under a range of agro-ecological conditions. Weather and price variability are other, risk-related, factors that farmers encounter.

Some labor and risk studies related to legume and fertilizer practices in maize-based cropping systems exist in Eastern and Southern Africa. For example, Rusinamhodzi (2015) clustered farmers in Zimbabwe based on resource endowments, including labor availability, and showed how digging planting basins (a reduced tillage method) increased labor demands. This increase seems in contrast with the desire to find technologies that simultaneously reduce labor demands and improve soil fertility. In Malawi, Thierfelder et al. (2015a) and Ngwira et al. (2012) focused on labor requirements, rather than labor availability, to highlight the labor-saving effects of alternative practices that fall under conservation agriculture, which includes growing legumes, such as cowpea or pigeon pea, in rotation with maize. Ortega et al. (2016) used choice experiments in central Malawi to show that labor demands are a major constraint to legume adoption. Studies have also shown that maize can have a greater labor-use efficiency than legumes such as groundnut (Franke et al. 2014), and that conservation agriculture can also raise labor-use efficiency compared with conventional agriculture (Thierfelder et al. 2015b). However, simultaneously examining labor supply and demand for different cropping systems can help highlight possible trade-offs associated with maize-legume integration at the household scale. For risk, studies considering weather variability in Malawi have shown that systems with legumes can increase yield stability (Ngwira et al. 2012, 2014). Some of these studies have shown how maize-legume rotations can affect profits. For example, Ngwira et al. (2012) and Ngwira et al. (2014) used average grain prices to compare profits in conventional and conservation agriculture systems. A cross-sectional study in Malawi showed that diversification of maize monoculture into a maize-legume system and using reduced tillage can increase crop yields and reduce downside risks (Kassie et al. 2015). Integrating legumes into maize monocultures and using fertilizer can increase productivity and partial profitability (Ngwira et al. 2012; Snapp et al. 2010). Ortega et al. (2016) urged additional research on risk and labor in the context of maize-legume systems.

Based on the above-mentioned studies, we see scope to improve our understanding of the economic, risk, and labor effects of planting legumes and applying fertilizer in maize-based cropping systems. Our study aims to provide useful insights into the potential labor, economic, and risk effects of changes in cropping practices for farmers in central Malawi. Many of these previous studies use a partial economic budgeting approach, which often only considers the gross value of production and associated financial costs. We complement the existing literature by considering the opportunity cost of labor in our economic profit calculations. We supplement studies that use cross-sectional household survey data by including a risk analysis based on variability in both grain yields and prices over time, thus adding a temporal dimension to trade-off analysis. We complement the above-mentioned studies on labor-use efficiency by examining labor balances. Our study aims to answer two questions:

1. How do different legume and mineral fertilizer practices affect productivity, labor use, profit, and risk?
2. Do farmers access enough agricultural labor to sustain a maize-groundnut rotation?

## Methods

### Background

Malawian farmers typically grow maize monoculture (continuous cropping of maize) often rotated or intercropped with legumes or sometimes rotated or intercropped with cassava or cash crops. Maize consistently occupies over 70% of cultivated land in Malawi, with groundnut the most commonly grown legume (FAO 2017). Fallows are rare in Malawi (Mungai et al. 2016). Farmers often apply fertilizer to their fields (Mungai et al. 2016). Farmers have a limited ability to use manure as an organic source of nitrogen because they keep minimal livestock.

Our study focused on the Golomoti Extension Planning Area of central Malawi. This area is in the lakeshore zone within the Dedza district and is approximately 500 m above sea level. Seasonal precipitation averaged 734 mm from 1989 to 2010, with a coefficient of variation (C.V., defined as the ratio between the standard deviation and average) of 0.16 suggesting a relatively stable precipitation. We characterized households in Golomoti with household survey data collected as part of the Africa Research In Sustainable Intensification for the Next Generation (Africa RISING) program (IFPRI 2015), which included 121 households. The survey was conducted in the summer of 2013, with data referring to crops grown between October 2012 and May 2013. The survey collected data on family size, grain yields, areas cultivated, and inputs and labor used for cropping activities (at the household-crop-activity level).

### Cropping system simulations

We used the Decision Support System for Agro-technology Transfer (DSSAT) model v4.5 (Jones et al. 2003) to simulate crop yields for 22 years from 1989 to 2010 to generate data on grain yields and nitrogen-use efficiency. Indicators (discussed in section 2.3) were calculated based on simulated data, household survey data, and price and cost data from secondary sources. We simulated four cropping systems, the first three are the continuous cropping of maize with differing nitrogen [N] fertilizer application rates:

1. Maize monoculture with no fertilizer applied (MM0—unfertilized);
2. Maize monoculture with 35 kg [N] ha^−1^ of urea fertilizer applied (MM35—moderately fertilized);
3. Maize monoculture with 69 kg [N] ha^−1^ of urea fertilizer applied (MM69—intensely fertilized); and
4. Maize-groundnut rotation with 35 kg [N] ha^−1^ of urea fertilizer applied to maize and 12 kg [N] ha^−1^ of urea fertilizer applied to groundnut (MG).

These systems matched those in a Golomoti field experiment reported in Smith et al. (2016). To match the protocol in the field experiment, each simulated system omitted the application of manure and removed 70% of crop residues from the field. The four cropping systems reflect a mix of current and alternative farmer practices and government recommendations. For example, the Malawian government recommends applying 69 kg [N] ha^−1^ to maize (Mungai et al. 2016).

We simulated yearly crop yields over time to help account for temporal weather variability and the cumulative effects of on-farm practices on productivity. We calibrated DSSAT with local data on soils, crop cultivar characteristics, and management of the crop(s) for the conditions of the study area. Model calibration data were taken directly from Smith et al. (2016). Soil data included, among others, soil texture (% sand, silt, and clay), soil % carbon and nitrogen, soil pH, Bray P (ppm), plant available water capacity (mm), and fraction of organic carbon in microbial biomass for each standard soil profile layer depth (0–15 centimeters (cm), 15–30, 30–60, 60–90, and 90–120 cm). Calibration data for crop cultivar and crop management included crop residue use, planting density, planting depth, fertilizer applied, and rotation. Genetic growth coefficients for our DSSAT simulations of maize variety SC403 included P1 195.4, P2 0.852, P5 809.1, G2 607.2, G3 8.11, and PHINT 31.74. Ruane et al. (2015) supplied the daily weather data.

The performance of DSSAT has been previously evaluated in maize-based systems in Eastern and Southern Africa (Ngwira et al. 2014). To evaluate DSSAT in our study, we compared simulated grain yields with observed grain yields reported in Smith et al. (2016) from the 2012–2013 and 2013–2014 growing seasons. This included experimental data on maize yields in MM0, MM69, and MG, and groundnut yields in MG. We calculated the normalized root mean squared error—a quantitative measure of the deviation of simulated data from observed data.

### Cropping systems indicators

In this study, cropping system refers to either maize monoculture or the maize-groundnut rotation. The field scale refers to the cropping system simulated in DSSAT on a per hectare basis. The household scale refers to the cropping system simulated in DSSAT on a per-farm-household basis. At the household scale, we allocated the available arable land to each of the simulated cropping systems with the total area planted equal to the observed area the household planted to maize and legumes. Household data in Golomoti suggest that farmers allocate approximately 80% of their arable land to either maize or legumes (Mungai et al. 2016). The indicators we calculated for each system (discussed in sections 2.3.1 and 2.3.2) related to caloric yields, nitrogen-use efficiencies, labor balances, profits, and risks. We calculated all the indicators at the field-scale level, except for labor balance, which we calculated at the household scale. Groundnuts can be sold either shelled or unshelled, and shelling increased both labor use and sales price. Thus, we considered five systems from an economic perspective: (1) MM0, (2) MM35, and (3) MM69 defined in section 2.2, and MG for (4) shelled groundnuts (MGS), and (5) unshelled (MGUS) groundnuts.

#### Productivity and labor indicators

We simulated grain yields (kg ha^−1^) from each crop in each system and calculated the caloric yield of each system (kcal ha^−1^). Maize contained 357 kcal 100 g^−1^ and groundnut contained 549 kcal 100 g^−1^ (FAO 1968). Nitrogen-use efficiency for maize was calculated as the ratio of grain yield to nitrogen fertilizer applied, which measures the partial factor productivity of nitrogen fertilizer.

We used household survey data to calculate agricultural labor demand in each system and household labor available (both in days^−1^ season^−1^ household^−1^). Equation (1) defines agricultural labor demand for each cropping system at the household scale (LD_*h,s*_). Season refers to one of the four (meteorological) seasons: summer (December, January, and February), autumn (March, April, and May), winter (June, July, and August), and spring (September, October, and November).

1 $$ {\mathrm{LD}}_{h,s}=\sum \limits_a^A\sum \limits_c^C{L}_{a,c,s,h}\times \left({\mathrm{MA}}_h+{\mathrm{LA}}_{\mathrm{h}}\right) $$

In Eq. (1), *L*_*a,c,s,h*_ is the reported days spent on each cropping activity (*a*), for each crop (*c*), in each season (*s*) by each household (*h*) in person-days ha^−1^. The observed household area (in ha) of maize is MA_*h*_ and legumes is LA_*h*_. The combined maize and legume area was, on average, 81% of the total 1.06 ha of reported arable land (TA_*h*_). Thus, we had an indicator of labor demand for the five economic systems at the household scale in each season (LD_*h,s*_). The household survey collected data on labor use for crop activities (*a*) including land preparation, weeding, herbicide application, fertilizer application, organic matter application, pest control, and harvesting and post-harvest activities. The survey asked labor demands for harvesting and post-harvest activities as a single value. Labor activities for maize and groundnut occur in different months. For maize, June–July: incorporation of residues (clearing), August–October: incorporation of residues and ridging, November–December: ridging, planting, weeding, and fertilizing, January–February: weeding and fertilizing, and March–April: harvest. For groundnut, May–July: harvesting and clearing, August–September: post-harvest, November–December: planting, and January–February: weeding. To differentiate labor used in each simulated system by the amount of fertilizer applied, we used farmer-reported time for fertilizer application and the quantity of fertilizer applied to calculate the household-specific time taken to apply 1 kg of fertilizer. The time spent per kilogram was then used to calculate labor use for the differing fertilizer quantities, which varied by system. Household data are for shelled groundnuts, the common practice for sales, although a market does exist for unshelled groundnuts. To differentiate labor used for shelled or unshelled groundnuts, shelling required 20 days ton^−1^ of groundnut grain (Waddington et al. 2007).

Equation (2) defines labor supply at the household scale for each season (LS_*h,s*_).

2 $$ {\mathrm{LS}}_{h,s}={\mathrm{LF}}_{h,s}\times \left(\left(30\times 3\right)-{\mathrm{OFL}}_{h,s}\right)\times \left(\frac{{\mathrm{MA}}_h+{\mathrm{LA}}_h}{{\mathrm{TA}}_h}\right) $$

In Eq. (2), labor supply was calculated by multiplying the reported available family labor per household (persons aged > 15 and < 65 years, LF_*h,s*_) by the days available to allocate to the simulated cropping system. Each working family member had 30 days month^−1^ to allocate to either the simulated cropping system, other farm activities, or off-farm work (OFL_*s*_). We calculated the days each household allocated to off-farm work, based on reported off-farm income and local wages. The days spent on other farm activities were proportional to the area allocated to crops other than maize and legumes, i.e., 19% of the average household’s total arable land (TA_*h*_). The remaining days in each month (30 minus other farm activities minus off-farm work) were available for each worker to allocate to the simulated cropping system. The difference between family labor availability (LS_*h,s*_) and total labor demand (LD_*h,s*_) provides an insight into which systems might have a labor deficit.

#### Economic and risk indicators

We calculated two measures of field-scale profit (US $ ha^−1^) in each year (*y*) for each system: financial profits (FP_*y*_) shown in Eq. (3) and economic profits (EP_*y*_) shown in Eq. (4).

3 $$ {FP}_y=\sum \limits_c^C\left({GY}_{c,y}\times {P}_{c,y}\right)-\left(\left({QF}_c\times F\right)+\left(\mathrm{Q}{S}_c\times {S}_c\right)\right) $$

4 $$ {EP}_y={FP}_y-\left(\sum \limits_a^A\sum \limits_c^C{L}_{a,c}\times w\right) $$

Financial profits equaled the value of grain production, a multiplication of the grain yield of *c* in *y* (GY_*c,y*_) and its market price (P_*c,y*_), minus associated financial costs. Financial costs equaled the sum of the unit cost of fertilizer (*F*) and fertilizer quantity applied (QF_*c*_), plus the sum of the unit cost of seed (*S*_*c*_) and the quantity of seed used (*QS*_*c*_). Our study used grain prices in Golomoti markets from 1989 to 2010, supplied by the Malawian Agricultural Market Information System (IFPRI 2013). Nitrogen fertilizer cost 0.67 US $ kg^−1^ (Franke et al. 2014). Seed costs were calculated from the household survey and were 0.44 US $ kg^−1^ for maize and 0.43 US $ kg^−1^ for groundnut. Economic profits were financial profits minus the opportunity cost of labor, the latter defined as the implicit value of labor computed based on labor used—*L*_*a,c*_ from Eq. (4)—and the daily wage (*w*). The wage was 1.33 US $ day^−1^ (Franke et al. 2014). We maintain that the household first used family labor to meet labor demand and, if the labor balances in section 2.3.1 identified a negative labor balance, the household hired labor to meet the deficit. Hired labor had a 20% higher wage than the local wage, to account for transaction costs. Price and cost data were adjusted for inflation with the Malawi Consumer Price index for a base year 2013 with an exchange rate of 1 US $ = 150 Malawian Kwacha. Therefore, grain prices varied each year, whereas seed costs, fertilizer costs, and wages were fixed over time in real inflation-adjusted US $. We calculated the net present value of economic profits in each system from 1989 to 2010 using a discount rate of 6% per year. Net present values incorporate both the timing and magnitude of economic costs and benefits, which is important if profits change over time.

We calculated four indicators for economic risk in each system: the stability of profits, the probability of returning a positive economic profit, the average of the lowest 10% of profits (Conditional Value at Risk), and the certainty equivalent. The C.V. was used to measure the stability of profits. Next, we calculated the probability of a system generating a positive economic profit. Third, the Conditional Value at Risk of the lowest 10% of possible economic profits was calculated to measure the downside risk of extreme loss associated with unfavorable events. The Conditional Value at Risk is the average of the lowest 10% of economic profits for all the simulated years. Finally, we used Eq. (5) to calculate the certainty equivalent (CE) of each system. The certainty equivalent is a risk-adjusted measure of profits, defined as the difference between expected profit (EP) and a risk premium (RP*)*, i.e., CE = EP *−* RP (Antle 1987). Here, EP is the average of yearly economic profits (EP_*y*_). The certainty equivalent represents the smallest amount of certain money a farmer is willing to receive to forgo an uncertain profit. We calculated certainty equivalents with the method in Lehmann et al. (2013).

5 $$ CE= EP-\left(\frac{1}{2}\times \frac{r}{EP}\times V\right) $$

In Eq. (5), *r* is the Arrow-Pratt relative risk aversion coefficient and *V* is the variance of field-scale economic profits, and here, variance is the square of the standard deviation. The analysis of certainty equivalents only considers systems with a positive average economic profit. Equation (5) implies constant relative risk aversion. We computed the certainty equivalent of each system with calculated values for expected profits and their variance, similar to Lehmann et al. (2013). We calculated the certainty equivalent for *r* equal to zero (indifference to risk) and *r* equal to one (moderate risk aversion).

## Results and discussion

### Household characterization

Table 1 summarizes the Golomoti household survey data. Households had on average 1.06 ha of arable land of which 53% was maize and 28% was legumes, with the remaining land planted to a variety of crops such as cotton and sweet potato. The legume area (as a percentage of all arable land) was comparable to the 30% at the national level (FAO 2017). Maize yields averaged 1.6 t ha^−1^ (C.V. = 0.7) compared to the national average of 2.1 t ha^−1^ in 2013 (FAO 2017). Maize yields in Malawi take on a wide range. For example, Tamene et al. (2016) report maize yields to range between 0.4 and 12 t ha^−1^ in Dedza district. About 91% of households used fertilizer. For maize, the fertilizer application rate ranged from zero to 157.9 kg [N] ha^−1^, with average rates in Table 1 similar to rates in Mungai et al. (2016). Limited off-farm earnings and livestock assets were reported, buttressing calls to improve crop productivity as a livelihood improvement strategy.

**Table 1:** Summary of household survey data

Indicators	Average	C.V.	Minimum	Maximum
Total arable land (ha^−1^)	1.06	0.64	0.10	3.24
Maize area (ha^−1^)	0.56	0.69	0.039	2.12
Legume area (ha^−1^)	0.30	0.94	0	1.62
Maize yield (kg ha^−1^)	1553.3	0.68	159.4	5208.3
Fertilizer applied among users (kg [N] ha^−1^)	43.2	0.91	10.2	157.9
Off-farm income ($)	36.7	1.39	0	297.1
Tropical livestock units (number)	0.41	1.35	0	2.70

Data are reported at the household level and based on 121 households. N represents nitrogen. One tropical livestock unit equals a 250-kg liveweight ruminant. *C.V.* coefficient of variation

The average labor used (person-days ha^−1^) was 217 (C.V. = 0.62) for maize and 355 (C.V. = 0.70) for groundnut. The observed labor used for maize and groundnut broadly concurs with other calculations of labor use in central Malawi, for example, Franke et al. (2014). Land preparation was a major use of time for both crops. For groundnuts, the average household spent 91 days ha^−1^ for weeding and 87 days ha^−1^ for harvesting and post-harvest activities, whereas, for maize, weeding used 61 days ha^−1^ and harvesting and post-harvest activities used 31 days ha^−1^.

### Productivity and labor indicators

**Fig. 2:**
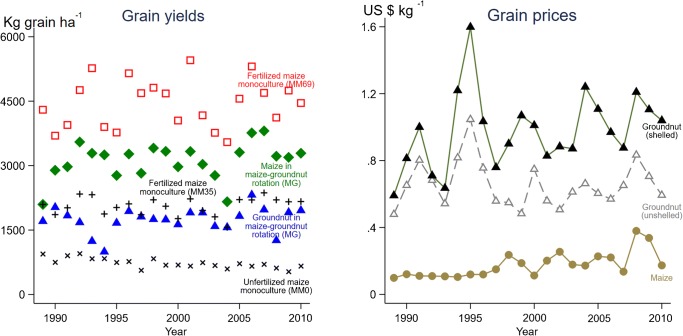
Simulated grain yields and inflation-adjusted maize and groundnut prices in Golomoti from 1989 to 2010. Fertilized maize monocultures had either 35 (MM35, black plus sign) or 69 (MM69, red hollow square) kg [N] ha^−1^ of urea applied. N represents nitrogen. The maize-groundnut rotation (MG) had 35 kg [N] ha^−1^ of urea applied to maize (green diamond) and 12 kg [N] ha^−1^ of urea applied to groundnut (blue triangle)

Comparing our simulated grain yields for two maize monocultures (MM0 and MM69) and the rotation (MG) with observed yields reported for these systems in Smith et al. (2016) produced a normalized root mean squared error of 14%. Simulated grain yields averaged 4447 kg ha^−1^ in the maize monoculture with 69 kg [N] ha^−1^ (C.V. = 0.13) (Fig. 2), with yields at least six times lower in the unfertilized maize monoculture. Simulated grain yields in the intensely fertilized maize monoculture (MM69) were almost double the national average, mainly because farmers across Malawi use, on average, less than the 69 kg [N] ha^−1^ applied in the simulations. Because farmers often apply some fertilizer (Table 1), their yields exceeded the simulated yields of unfertilized maize monoculture (MM0). Maize with 35 kg [N] ha^−1^ grown in rotation with groundnut (MG) had an average simulated yield of 3114 kg ha^−1^ (C.V. = 0.14), which was approximately 1 t ha^−1^ more than the 2063 kg ha^−1^ yield in the maize monoculture with 35 kg [N] ha^−1^ (MM35). Two factors helped explain the 1 t ha^−1^ yield benefit in the rotation. First, groundnut’s average biological nitrogen fixation rate of 117 kg [N] ha^−1^ helped increase the nitrogen content of soil in the rotation. Despite, in general, much of the nitrogen fixed by legumes being removed from the system in high-protein seed, a net residual contribution of fixed nitrogen to the nitrogen content of soil often exists. Our simulated nitrogen fixation rate for groundnut was within the range reported in other Malawi studies (Mhango et al. 2017), which were broadly comparable to our study. Second, in our study, we retained 30% of crop residues (section 2.2), including the green residues from groundnut. Green residues from groundnut often contain large amounts of nitrogen at harvest and can supply more nitrogen for subsequent crops and other grain legumes such as soybean. Consequently, the simulated nitrogen uptake by maize averaged 77 kg [N] ha^−1^ y^−1^ in the rotation (MG) and 58 kg [N] ha^−1^ y^−1^ in the moderately fertilized monoculture (MM35).

In Golomoti, groundnut prices exceeded maize prices, and maize prices had a higher C.V. than groundnut prices (Fig. 2). Shelled groundnut prices exceeded unshelled groundnut prices. The C.V. for maize prices was 0.43, exceeding the C.V. of the most variable groundnut price, 0.23. The correlation coefficient between maize grain price and groundnut (shelled) price was 0.24, and the correlation coefficient was − 0.08 between maize grain price and groundnut (unshelled) price.

The simulated caloric yield of 15.9 × 10^6^ kcal ha^−1^ in the intensely fertilized maize monoculture (MM69) was 54% greater than the 10.3 × 10^6^ kcal ha^−1^ yield in the maize-groundnut rotation (MG), and was greater than the unfertilized monoculture (2.6 × 10^6^ kcal ha^−1^) and the moderately fertilizer monoculture (MM35) (7.4 × 10^6^ kcal ha^−1^). The maize-groundnut rotation used more labor (281 days ha^−1^) than the monocultures (< 223 days ha^−1^), mainly because labor used for groundnut exceeded that for maize (section 3.1). Nitrogen-use efficiency was highest in the maize-groundnut rotation (89 kg grain kg [N] fertilizer^−1^), and declined as fertilizer application rates rose (65 kg grain kg [N] fertilizer^−1^ in MM69).

**Fig. 3:**
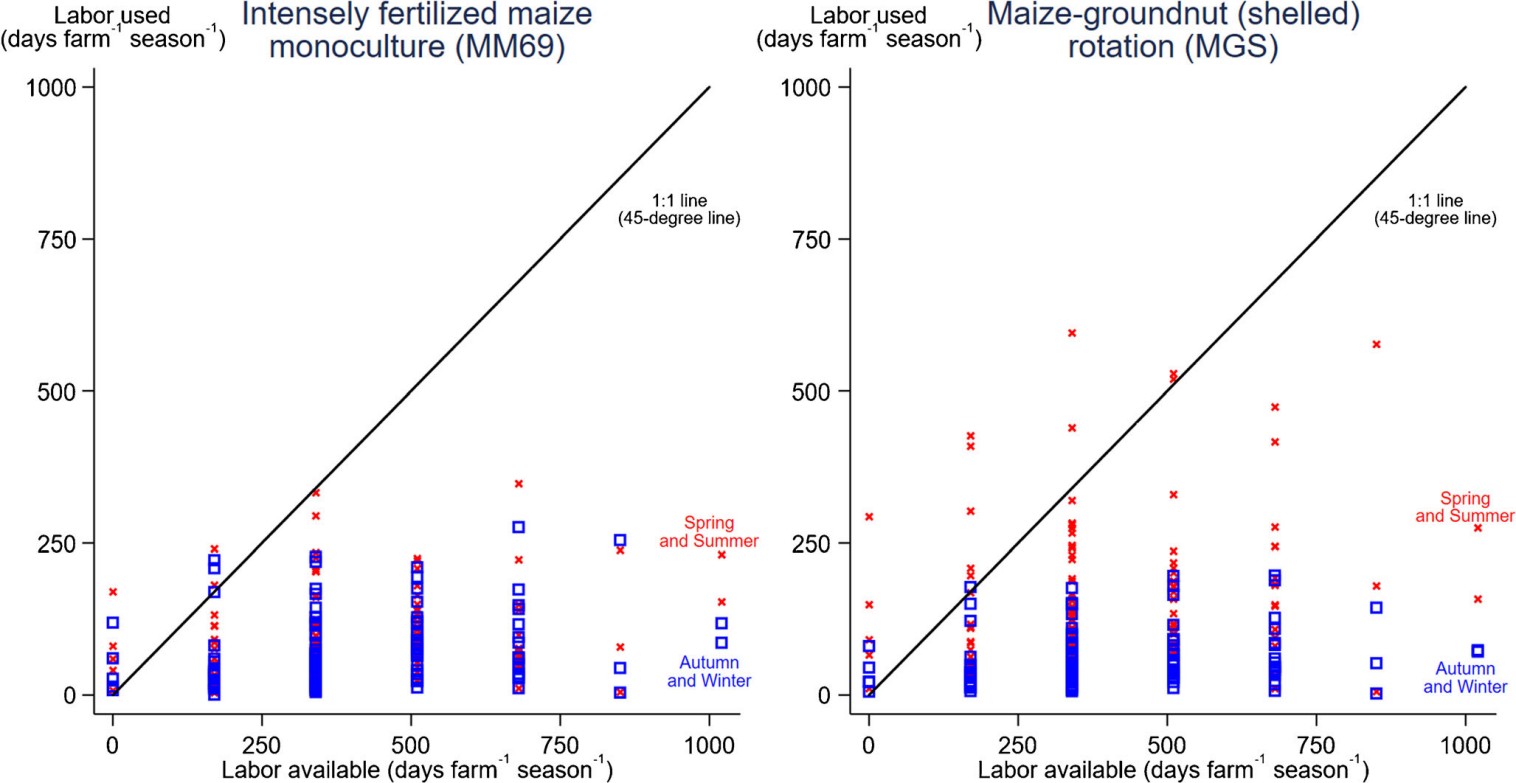
Seasonal labor use and availability for Golomoti farmers. Intensely fertilized maize monoculture (MM69) has 69 kg [N] ha^−1^ of urea applied. The rotation (MGS) has 35 kg [N] ha^−1^ of urea applied to maize and 12 kg [N] ha^−1^ of urea applied to groundnut. Groundnuts were sold shelled (shelled rotation). N represents nitrogen. Results reported for two aggregated time periods: Spring and Summer (red cross) (September to February), and Autumn and Winter (blue hollow square) (March to August)

Depending on the system examined, some households had labor use exceeding family labor supply (Fig. 3). More households incurred a labor deficit in the maize-groundnut rotations, compared with the maize monoculture (MM69) given that the former system required more labor than the latter (Section 3.1). Each year, the average household had 7 days in the maize monoculture (MM69) and 17 days in the maize-groundnut rotation (MGS) for which family labor supply was insufficient to meet labor requirements. The maize-groundnut rotations had the highest percentage of households with a negative labor balance during summer and spring. For example, if each surveyed household used one of the maize monocultures, 5% of households would have a labor deficit in spring, compared with 12% of households who used the maize-groundnut rotation. Labor dynamics add another complexity to the economics of integrating legumes into maize systems. Ngwira et al. (2012), Franke et al. (2014), and Thierfelder et al. (2015b) calculated labor-use efficiency to better understand the productivity of maize-legume systems. Ngwira et al. (2012) and Thierfelder et al. (2015a) found that maize monoculture can use slightly less labor than a maize-legume system. Ngwira et al. (2012) also showed maize monocultures can have a higher labor productivity (kg grain per day worked^−1^) than maize-legume systems. Here, we add to these productivity-focused studies by illustrating potential bottlenecks between labor required and available at the household scale (Fig. 3).

### Economic and risk indicators

Table 2 summarizes how different practices affected simulated economic and risk indicators. Costs were the highest in the intensely fertilized maize monoculture (MM69), attributable to the cost of fertilizer. Malawian farmers are often sensitive to changes in the cost of fertilizer (Komarek et al. 2017), with cost changes posing a threat to the attractiveness of fertilizer use. Shelling increased labor use for groundnut, which increased the implicit cost of labor. The unfertilized maize monoculture (MM0) was the least profitable system, having a negative economic profit in all years, indicating that the value of crop production was less than the total cost of fertilizer, seed, and implicit labor. The maize monocultures had greater downside risks than the maize-groundnut rotations, with the Conditional Value at Risk negative in the maize monocultures but positive in the maize-groundnut rotations. The most profitable system (MGS in Table 2) produced less calories than the intensely fertilized maize monoculture (MM69) (section 3.2). Profit in fertilized maize monocultures is often similar to or lower than profit in maize-legume systems, but highly context specific (Thierfelder et al. 2015b; Ngwira et al. 2012). In our study, we examined average yearly economic profits and also examined net present values. The intensely fertilized maize monoculture (MM69) had a 19% lower net present value than the maize-groundnut (unshelled) rotation (MGUS), despite MM69 and MGUS having similar economic profits—357 US $ ha^−1^ in MM69 and 355 US $ ha^−1^ in MGUS. The cumulative agronomic benefits of integrating legumes into maize systems, combined with changes in prices, translated into these net present values. Yield is only one indicator farmers consider when evaluating alternative crop practices and, in our study, the system that produced the most calories generated less profit than the most profitable system. Franke et al. (2014) showed that maize produces a higher caloric yield than groundnut, which in turn has higher caloric yield than soybean. Franke et al. (2014) showed that replacing half of a simulated farm’s maize and soybean area with groundnut can slightly increase caloric production, possibly because soybean has a lower caloric yield than groundnut. This replacement ultimately increased profits. Our study had systems with differing practices (i.e., fertilizer rates), but the broad context was similar. In addition, we found that risk indicators differed across the systems.

**Table 2:** Economic and risk indicators for the simulated cropping systems

Indicator	MM0	MM35	MM69	MGUS	MGS
Average financial input cost (US $ ha^−1^)	6.76	37.9	68.1	32.1	32.1
Average cost of labor (US $ ha^−1^)	341.7	351.2	359.4	452.3	460.5
Average financial profit (US $ ha^−1^)	116.6	326.6	716.5	807.7	1086.7
Coefficient of variation of financial profit	0.37	0.53	0.50	0.23	0.26
Average economic profit (US $ ha^−1^)	− 225.0	− 24.6	357.1	355.3	626.2
Standard deviation of economic profit	43.6	173.4	361.3	182.2	280.6
Coefficient of variation of economic profit	.	.	1.01	0.51	0.45
Net present value (US $ ha^−1^)	− 2810	− 788	3246	3855	6733
Probability of positive economic profit (%)	0	31.8	90.9	100	100
Conditional Value at Risk (US $ ha^−1^)	− 267.2	− 191.8	− 12.3	28.8	97.4
Risk premium (RP) (US $ ha^−1^)	.	.	182.8	46.7	62.9
Certainty equivalent (CE) (US $ ha^−1^)	.	.	174.3	308.6	563.3

MM0 indicates unfertilized maize monoculture. Fertilized maize monoculture with 35 (MM35) and 69 (MM69) kg [N] ha^−1^ of urea. Maize-groundnut (shelled) rotation (MGS) and maize-groundnut (unshelled) rotation (MGUS) had 35 kg [N] ha^−1^ of urea applied to maize and 12 kg [N] ha^−1^ of urea applied to groundnut. Groundnuts were sold shelled (MGS) or unshelled (MGUS). N represents nitrogen. RP and CE use an Arrow-Pratt relative risk aversion coefficient of 1

The maize-groundnut (unshelled) rotation (MGUS) had similar average economic profits and approximately a 50% lower standard deviation in economic profit (calculated as variability over simulated years) compared with intensely fertilized maize monoculture (MM69) (Table 2). The maize-groundnut rotation had a lower variance of economic profits partly because groundnut prices had a lower C.V. than maize prices. In addition, the caloric yield of the maize-groundnut rotation had a slightly lower C.V. than the C.V. of intensely fertilized maize monoculture. Growing two crops as opposed to a monoculture reduced the C.V. of profits, given the minimal level of correlation between maize and groundnut prices (section 3.2). The unfertilized maize monoculture had negative profits in all 22 years and even MM35 had a 32% chance of negative profits, highlighting monoculture can be unprofitable—as also shown in Franke et al. (2014). The maize-groundnut rotations had more years of positive profits (100%) than the intensely fertilized maize monoculture (MM69) (91%). When we accounted for risk aversion, both maize-groundnut rotations had a higher certainty equivalent (risk-adjusted profit) than the intensely fertilized maize monoculture. When the Arrow-Pratt relative risk aversion coefficient was equal to one, the certainty equivalent of the maize-groundnut (unshelled) rotation was US $ 309 ha^−1^and was US $ 174 ha^−1^ in the intensely fertilized maize monoculture (MM69) (Table 2). However, economic profits in the rotation were similar to profits in MM69 (Table 2). Including risk aversion resulted in the maize-groundnut rotation providing higher risk-adjusted profits (the certainty equivalent) than under risk neutrality. Providing advice based on risk neutrality generated different systems rankings than providing advice based on risk aversion. Under risk neutrality, the intensely fertilized maize monoculture and the maize-groundnut (unshelled) rotation had similar average profits. However, under risk aversion, the intensely fertilized maize monoculture generated a lower certainty equivalent compared to the maize-groundnut (unshelled) rotation. This lower certainty equivalent finding complements Gandorfer et al. (2011), who reported that risk analyses rarely compare the certainty equivalents for risk-averse and risk-neutral strategies. Taken together, our results suggest that maize-groundnut rotations can reduce economic risks compared with maize monocultures.

## Conclusions

We examined how simulated changes in crop practices altered the performance of maize-based cropping systems with data from central Malawi. Four cropping systems were simulated: unfertilized maize monoculture, two fertilized maize monocultures, and a maize-groundnut rotation. Our study demonstrated some of the trade-offs and synergies that existed among indicators of different cropping systems related to caloric yields, labor balances, profits, and risks. Overall, the maize-groundnut rotation, relative to the intensely fertilized maize monoculture, yielded a higher nitrogen-use efficiency, was more profitable, had a higher net present value, and had reduced risks. However, the rotation produced lower caloric yields and required more labor, relative to the intensely fertilized maize monoculture. Because of differences in labor requirements, more households incurred a labor deficit in the maize-groundnut rotation, compared with those under the maize monocultures.

Hybrid studies, such as ours, that combine secondary data from field experiments with primary household survey data and simulation modeling are complements—not substitutes—to field experiments. Simulations can help identify possible trade-offs and synergies associated with counterfactuals of changes in crop practices on system indicators, which might be a challenge to demonstrate in an experimental setting. Highlighting these trade-offs and synergies helps build evidence for policy dialogs on how to support the sustainable development of cropping systems for labor- and resource-limited farmers.

### Data availability statement

The replication materials, including datasets and code, for the current study are available in the Dataverse repository, 10.7910/DVN/ILVSXJ.
